# A Manual of Pathological Anatomy

**Published:** 1855-04

**Authors:** 


ART. VIII.—*A Manual of Pathological Anatomy.* By C. HANDFIELD JONES, M. B., F. R. S., Fellow of the Royal College of Physicians, etc., and EDWARD H. SIEVEKING, M. D., F. R. C., etc. First American Edition Revised. Philadelphia: Blanchard and Lea. 1854.

A handsome octavo of 700 pages, fully illustrated. In its preparation the authors have divided the subject between them; Dr. Jones undertaking the morbid states of the blood, textural changes, new formations, parasites, together with the pathological anatomy of the alimentary canal, the urinary apparatus, and of the joints; while Dr. Sieveking has the pathology of the nervous system, of the organs of circulation, respiration, the female organs of generation, and of the osseous system.

Our space, as well as the difficulty of analyzing a book which treats of multitudinous subjects, prevents any lengthened critique. From the casual examination we have given we are inclined to regard it as a *text-book*, not minute, deeply learned, or abstruse in its descriptions or speculations; but plain, rational, and intelligible, such a book as the practical man needs for daily reference. For this reason it will be likely to be largely useful, as it suits itself to those busy men who have little time for minute investigation, and prefer a summary to an elaborate treatise.

For sale by MILLER, ORTON & MULLIGAN. H.

